# Prevalence of Road Risk Behaviors and Associated Factors Among Undergraduate College Students in Delhi: Findings From the Health Risk Behavior Survey

**DOI:** 10.7759/cureus.28123

**Published:** 2022-08-17

**Authors:** Mohit Goyal, Anita Verma

**Affiliations:** 1 Community Medicine, Vardhman Mahavir Medical College and Safdarjung Hospital, Delhi, IND

**Keywords:** drinking and driving, helmet, mobile phone, road safety, road risk, youth, college students

## Abstract

Background: Youth constitute one of the most vulnerable groups for practicing risky road behaviors. Road traffic accidents (RTAs) are one of the leading preventable causes of disability and mortality among children and young adults across the globe.

Objective: To estimate the prevalence of road risk behaviors among college students of Delhi and to determine the factors associated with it.

Methods: A cross-sectional study was conducted from January 2020 to September 2021 across five administrative zones in Delhi, India. Six hundred seventy-five undergraduate college students were selected across five colleges using stratified random sampling.

Results: The mean age of the study participants was 19.62 years (S.D. = ± 1.328). Among the study participants, more females (52.6%) were present than males (47.4%). Almost one-fifth of the participants reported not wearing a seat belt while driving or riding in a car during the past 30 days. Some 37.2% of the participants reported using a mobile phone while driving a car within the past 30 days. The prevalence of riding in a car driven by a person after drinking alcohol was 17.4%. Similarly, the prevalence of drinking and driving was 17.2% amongst the study participants. The prevalence of not wearing a helmet while driving or riding a two-wheeler vehicle was 42.6%. The overall prevalence of risky road behaviors was 16.7%. The multivariate analysis results revealed the odds of road risk behaviors to be significantly higher among those who were alcohol users (adjusted odds ratio, aOR=7.3, confidence interval, CI=3.8-13.8), substance abusers (aOR=2.4, CI=1.4-4.3), and those belonging to rural areas (aOR=4.2, CI=2.4-7.3).

Conclusion: The prevalence of road safety-related risky health behaviors was high among the study participants. The significant road-risk behaviors were not wearing a helmet while riding or driving a two-wheeler vehicle, texting or talking while driving, and driving a car under the influence of alcohol or drugs.

## Introduction

Deaths and injuries resulting from road traffic accidents (RTAs) remain a serious public health problem worldwide. According to Global Status Report on Road Safety 2018, released by the World Health Organization (WHO), 1.35 million people die, and another 20-50 million sustain injuries from RTAs globally each year [1]. RTAs are the eighth leading cause of death for all age groups and are the primary cause of death among children and young adults between 5 and 29 years of age [1]. More than 90% of the world's road traffic fatalities occur in developing countries [2]. India has the highest number of road accident-related deaths, accounting for 11% of all road accident-related fatalities worldwide [3]. According to the 2019 Road Accident Report, 449,002 accidents occurred in India in 2019, resulting in 151,113 deaths and 451,361 injuries [4]. Although there has been a 0.2% decrease in deaths due to RTAs compared to previous years, the overall number of incidents remains alarmingly high. These RTAs not only cause significant morbidity and mortality but they are also responsible for considerable economic losses to individuals, their families, and countries. Risky behaviors such as over-speeding, drinking and driving, non-usage of helmets and seatbelts while driving, and distracted driving using mobile phones are some of the significant risk factors for these RTAs [2, 4]. Youth constitute one of the most vulnerable groups to practice these risky behaviors. More young people between 15 and 29 years die from road crashes than from HIV/AIDs, malaria, tuberculosis, or homicide annually [2, 5]. Motor vehicle accidents, in particular, constitute an immense burden in terms of injury and death amongst adolescents and youth. In 2019, over 115,000 adolescents died from RTAs worldwide [5]. The behaviors established during adolescence and youth tend to persist throughout their lifetime. As a result, preventing RTAs is critical to raising awareness and implementing appropriate road safety measures. In order to plan and design preventive programs and interventions to raise awareness, baseline data on the magnitude of the problem must be collected. As there is a dearth of literature on this topic in India, as a result, the current study seeks to determine the prevalence of risky road behaviors among undergraduate college students in Delhi and the factors associated with this behavior. The findings can assist traffic safety and public health professionals in designing, selecting, tailoring, and implementing effective strategies that can significantly reduce risk behaviors, thereby preventing future crashes, injuries, and deaths among this age group. 

## Materials and methods

An observational, descriptive cross-sectional study was conducted from January 2020 to September 2021 in New Delhi, India. The study sample consisted of undergraduate college students in their first, second, or third year of graduation at the selected colleges affiliated with a large public university in Delhi. The study's sampling frame comprised colleges and students who met the following eligibility criteria.

Inclusion criteria:

1. Co-educational colleges affiliated with the largest public university,

2. All students currently enrolled as full-time students in undergraduate programs in the selected colleges of Delhi.

3. Students who either rode or drove a car or a two-wheeled motor vehicle in the past 30 days.

Exclusion criteria:

1. Colleges providing education for students with special needs, professional educational programs like College of Nursing, Medicine, and Dental Colleges.

2. Students who were enrolled as part-time students or enrolled in correspondence or distance-learning programs.

3. International students enrolled in exchange programs.

Sample size

Based on a study by Swain et al. (2014) [6], the prevalence of mobile phone use while driving was 17% among their study participants. Using this prevalence at a 95% confidence level, a 3% absolute error, and a 10% non-response rate, the calculated sample size for the study was 675.

Sampling technique

**Figure 1:**
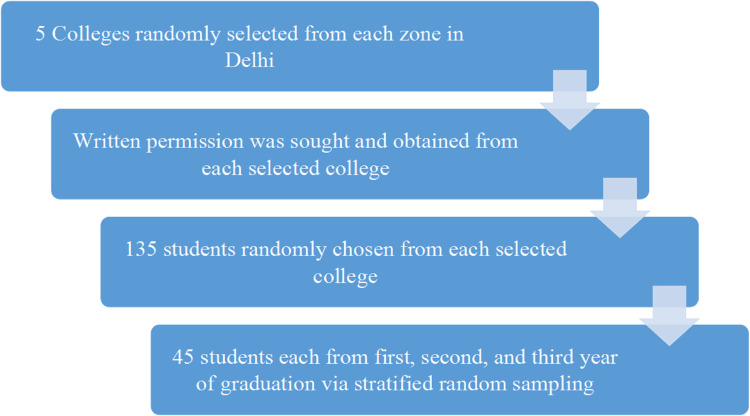
Schematic representation of the selection of study participants.

A two-stage stratified random sampling procedure was used. Firstly, a list of all eligible colleges affiliated with Delhi's largest public university was compiled based on the zones. Then, five colleges were chosen randomly from each zone (North, South, East, West, and Central Delhi) using a stratified random sampling technique; one college from each zone was chosen using computer-generated random numbers. Then, from the respective institutions, a list of all the students enrolled according to their graduation year was obtained. Given that the students are divided into different subject streams and years, 135 students were chosen at random from each college using stratified random sampling; equal numbers of students were chosen at random from each of the three years (45 each from the first, second, and third years of graduation) (Figure 1).

Study tool

A pre-designed, pre-tested, and self-administered questionnaire was used for data collection in the study.

*Socio-demographic data*: The first part of the questionnaire dealt with demographic details of the study participants which included age, gender, religion, parent’s education, place of permanent residence, current residential status, current academic status, current tobacco use, alcohol use, and substance abuse.

*Road risk behavior*: The second part assessed road safety behaviors based on the YRBS-2019 questionnaire developed by the Centers for Disease Control and Prevention (CDC) and was used to conduct a national survey amongst high school students in the United States biennially [7].

In order to assess road risk behaviors, the following questions were asked:

1. During the past 30 days--- “never," "rarely," or "sometimes" wearing a seat belt when sitting in the front seat of a car while riding or driving;

2. During the past 30 days --- “never”, “rarely”, or ― ”sometimes” wearing a helmet while riding or driving a two-wheeler vehicle; 

3. Drinking and driving (a car or motorcycle) on one or more occasions in the previous 12 months;

4. Texting or talking on the phone in the past 30 days while driving a car.

These six risk behaviors were divided into three domains: risky riding, risky driving, and helmet use. To compute the overall prevalence, any person having the simultaneous presence of three or more risk factors across all the three domains was considered at risk. If a participant was ineligible on any one of the domains (for example, did not drive a car but rode in one or used a two-wheeler), then the risk was assessed for only the behaviors practiced; the presence of one or more risk factors was considered to be risky for these participants who either did not ride or drive in the previous 30 days.

Operational definitions used

*Current Tobacco User*: those who used any tobacco products at any time (one or more days) in the last 30 days.

*Current Alcohol User*: students who currently drank alcohol (at least one drink of alcohol on at least one day during the 30 days before the survey)

*Substance User*: considered to be any student who used marijuana (cannabis) or any other illicit drugs at least once in their lifetime.

Data analysis

The data collected were entered in a Microsoft Excel spreadsheet in coded form. Relevant variables were created, and suitable coding was done for each. The data were analyzed using the licensed version of IBM Statistical Package for Social Sciences (SPSS version 21, IBM Corp., Armonk, NY). All the variables were analyzed using descriptive statistics; categorical data were expressed in either frequency or proportions, and continuous data were expressed as mean with standard deviation (SD). Bivariate analysis was done using the Chi-square test (or Fisher’s exact test wherever applicable) and binary logistic regression to determine the association between road risk behaviors and other possible risk factors. Multiple logistic regressions were applied to analyze the relationship if any.

Ethical considerations

Clearance for the study was taken from the Institutional Ethics Committee. Written permission was taken from the Principals of each selected college. Written informed consent was taken from the participants, and personal information was kept strictly confidential. The survey was completely anonymous, and there is no way of linking the responses back to the students or the college.

## Results

Table 1 shows that the mean age of the study participants was 19.62 years (SD = ± 1.328). Almost half (48.9%) of the study participants belonged to the age group of 17-19 years, and 51.1% belonged to the age group of 20-23 years. Among the study participants, females (52.6%) were more than males (47.4%). Three-fourths (74.8%) of the study participants belonged to urban areas, while almost a quarter (25.2%) originally belonged to rural areas. Mothers of about two-thirds of the participants (63.3%) were either graduates or postgraduates, whereas 36.7% of the participants reported their mothers not being graduates. More than one-third (34.5%) of the participants reported that their fathers did not go to college, while two-thirds (65.5%) reported that their fathers had a graduate or postgraduate degree. Almost one-third of the participants were pursuing Arts (34.5%), Science (33.8%), and Commerce (31.7%) related courses. Based on self-reported academic performance, most study participants (75.1%) reported having grades more than 70%, whereas a quarter (24.9%) reported their grades as less than 70%. 

**Table 1:** Distribution of study participants according to socio-demographic characteristics (N=675). SD, standard deviation

Characteristics	Number (N) (%)
1. Age group* (years)	
17-19	330 (48.9)
20-23	345 (51.1)
* Mean age = 19.62 years; SD = ± 1.328; Max = 23 Min = 17; Range = 6	
2. Gender	
Female	355 (52.6)
Male	320 (47.4)
3. Place of Birth	
Rural	170 (25.2)
Urban	505 (74.8)
4. Religion	
Hinduism	570 (84.4)
Islam	59 (8.7)
Christianity	28 (4.1)
Others	18 (2.7)
5. Mother's Educational Status	
Graduate and above	427 (63.3)
Not a Graduate	248 (36.7)
6. Father's Educational Status	
Graduate and above	442 (65.5)
Not a Graduate	233 (34.5)
7. Year of College	
1^st^ Year	225 (33.3)
2^nd^ Year	225 (33.3)
3^rd^ Year	225 (33.3)
8. Educational Stream	
Science	228 (33.8)
Commerce	214 (31.7)
Arts	233 (34.5)
9. Academic Performance	
Grades ≥ 70%	507 (75.1)
Grades < 70%	168 (24.9)
10. Current Smoking Status	
Yes	137 (20.3)
No	538 (79.7)
11. Current Alcohol Use	
Yes	168 (24.9)
No	507 (75.1)
12. Ever Substance Use	
Yes	131 (19.4)
No	544 (80.6)

In Table 2, out of the total study participants, nearly one-fifth (19.7%) of the participants who rode in a car during the past 30 days reported that they never had, rarely, or sometimes worn a seat belt. In contrast, the majority (80.3%) reported wearing a seat belt on most occasions during the month preceding the survey. Almost half of the participants (48.9%) drove a car in the previous month, with one-fifth (19.2%) not wearing a seat belt most of the time or always while driving. Non-seat belt use was prevalent amongst just about one-fifth of the participants while riding (19.7%) and driving (19.2%). The prevalence of riding in a car driven by a person under the influence of alcohol was found to be 17.4% amongst the study subjects.

**Table 2:** Prevalence of risky road behavior amongst the study participants. #Values are not mutually exclusive **For those that drove and rode in the past 30 days ***For those who either did not ride or drive ##Co-occurrence of three or more behaviors out of the six mentioned in the above domains was considered for the overall risky road-related behavior prevalence. If a participant was eligible for only one or two domains then occurrence of a single event in the past 30 days was considered to be risky.

Behavioral characteristics		Number (%)
I. Risky Riding Behavior (n=615)*^#^		
1. Wore a seatbelt when riding		
Never, rarely, or sometimes wore a seatbelt		128 (19.7)
Most of the time or always wore a seatbelt		487 (80.3)
2. Rode with a driver who had drunk alcohol		
Yes		107 (17.4)
No		508 (82.6)
*60 study participants did not ride in a car or motor vehicle in the past 30 days		
II. Risky Driving Behavior (n=323)*^#^		
1. Wore seat belt when driving		
Never, rarely, or sometimes wore a seatbelt		62 (19.2)
Most of the time or always wore a seatbelt		261 (80.8)
2. Drove car at least once after drinking alcohol		
Yes		58 (17.9)
No		265 (82.1)
3. Texted, talked, or e-mailed when driving		
Yes		120 (37.2)
No		203 (62.8)
*352 study participants did not drive a car in the past 30 days		
III. Helmet Related Risky Behavior (n= 337)^#^		
Wore a helmet during riding/driving a two-wheeler vehicle		
Never, rarely, or sometimes		144 (42.6)
Most of the time or always		194 (57.4)
*338 participants did not drive or ride a two-wheeler vehicle where a helmet had to be worn		
Overall Risky Road Related Behavior Prevalence^##^		
Risky Behavior	Number (%)	
Three or more risk factors present**	82(12.1)	
One or more risk factors present***	31(4.6)	
Risky behavior absent	562(83.3)	

Similarly, drinking and driving were found to be prevalent amongst 17.2% of the study participants. Nearly half (42.6%) of the 337 participants who rode a two-wheeled vehicle in the previous month reported not wearing a helmet always or on most occasions (Table 2). When all six risky behaviors in this domain were combined, nearly one-fifth (16.7%) of the participants practiced risky road behaviors (Table 2).

Table 3 shows that, on analyzing risky road behaviors of study participants with socio-demographic characteristics, it was observed that age, gender, belonging to the rural area, parent's educational status, current living status, academic year, and academic performance were found to be significantly associated with the practice of risky road behaviors, amongst the study participants (p<0.05). A proportion of students practicing other risky lifestyle behaviors such as drinking alcohol, substance abuse, and smoking also displayed a significantly higher prevalence of risky road-related behaviors than non-drinkers, non-abusers, and non-smokers (p < 0.05). However, the stream of the study had no statistically significant association with indulging in road risk behaviors (p > 0.05).

**Table 3:** Association of independent factors with risky road behavior amongst study participants (N=675). *Significant association with p-value (< 0.05); #Chi-square test

Characteristic	Risky road behavior		Total N (%)	p-value
	NO n (%)	YES n (%)		
I. Age group ( in completed years)				<0.01*^#^
17-19	294(89.1)	36(10.9)	330(100)	
20-23	268(77.7)	77(22.3)	345(100)	
II. Gender				<0.01*^#^
Female	312(87.9)	43(12.1)	355(100)	
Male	250(78.1)	70(21.9)	320(100)	
III. Place of origin				<0.01*^#^
Rural	121 (71.2)	49(28.8)	170(100)	
Urban	441 (87.3)	64(12.7)	505(100)	
IV. Mother’s educational status				<0.01*^#^
Not a graduate	189(76.2)	59(23.8)	248(100)	
Graduate and above	373(87.4)	54(12.6)	427(100)	
V. Father’s educational status				<0.01*^#^
Not a graduate	174(74.7)	59(25.3)	233(100)	
Graduate and above	388(54)	54(12.2)	442(100)	
VI. Year of study				<0.01*^#^
1st Year	202(89.8)	23(10.2)	225(100)	
2nd Year	184(81.8)	41(18.2)	225(100)	
3rd Year	176(78.2)	49(21.8)	225(100)	
VII. Subject of study				0.328#
Science	183(80.3)	45(19.7)	228(100)	
Commerce	181(84.6)	33(15.4)	214(100)	
Arts	198(85)	35(15)	233(100)	
VIII. Grades				<0.01*^#^
≥70%	440(86.8)	67(13.2)	507(100)	
≤70%	122(72.6)	46(27.4)	168(100)	
IX. Living with Parents				<0.01*^#^
Yes	452(88.5)	59(11.5)	511(100)	
No	110(67.1)	54(32.9)	164(100)	
X. Current Smoking Status				<0.01*^#^
Yes	89(65)	48(35)	137(100)	
No	473(87.9)	65(12.1)	538(100)	
XI. Current Alcohol Use				<0.01*^#^
Yes	101(60.1)	67(39.9)	168(100)	
No	461(90.9)	46(9.1)	507(100)	
XII. Ever Substance Use				<0.01*^#^
Yes	80 (61.1)	51(38.9)	131(100)	
No	482(88.6)	62(11.4)	544(100)	

A logistic regression analysis was performed for factors independently associated with road risk behaviors by chi-square test to determine independent determinants of road risk behaviors among college students. Significantly associated variables identified in univariate analysis with p > 0.2 were included in the model. The multivariate analysis results revealed odds of road risk behaviors to be significantly higher among alcohol users (adjusted odds ratio, aOR=7.3, confidence interval, CI=3.8-13.8), substance abusers (aOR=2.4, CI=1.4-4.3), and those belonging to rural areas [aOR= 4.2, CI=2.4-7.3]. However, being a current smoker (aOR=1.5, CI=0.8-3.2) did not significantly increase road-risk-related behavior odds. Moreover, age, gender, year of study, the field of study, grades, educational qualification of parents, and current living status of the students did not show any significant increase in the odds of higher risk-road behaviors respectively (Table 4).

**Table 4:** Bivariate and multivariate logistic regression analysis output for factors associated with risky road behavior (N=675). OR, odds ratio; CI, confidence interval

Variable	Total N (%)		Unadjusted OR; 95%CI	p-value	Adjusted OR; 95%CI	p-value
I. Age group						
17-19	330(48.9)		Reference		Reference	
20-23	345(51.1)		2.3(1.5-3.6)	<0.01*	1.3(0.7-2.6)	0.43
II. Gender						
Female	355(52.6)		Reference		Reference	
Male	320(47.4)		2(1.3-3)	<0.01*	1.4 (0.8-2.3)	0.15
III. Place of Birth						
Rural	170(25.2)		2.8(1.8-4.3)	<0.01*	4.2 (2.4-7.3)	<0.01*
Urban	505(74.8)		Reference		Reference	
IV. Educational Status of Mother						
Graduate or above		427(63.3)	Reference		Reference	
Not a graduate		233(36.7)	2.2(1.4-3.2)	<0.01*	0.86 (0.42-1.8)	0.67
V. Educational status of Father						
Graduate or above		442(65.5)	Reference		Reference	
Not a graduate		233(34.5)	2.4(1.6-3.7)	<0.01*	1.054(0.5-2.2)	0.90
VI. Year of Study						
1st Year		225(33.3)	Reference		Reference	
2nd Year		225(33.3)	1.9(1.1-3.3)	.02*	1.37 (0.7-2.7)	0.38
3rd Year		225(33.3)	2.5(1.4-4.1)	<0.01*	1.44 (0.7-3.2)	0.37
VII. Grades						
≥70%		508(75.3)	Reference		Reference	
≤70%		167(24.7)	2.5(1.6-3.8)	<0.01*	0.8(0.4-1.4)	0.51
VIII. Living with Parents						
Yes		511(75.7)	Reference		Reference	
No		164(24.3)	3.7(2.5-5.7)	<0.01*	0.7(0.4-1.2)	0.19
IX. Current Smokers						
No		538(79.7)	Reference		Reference	
Yes		137(20.3)	3.9(2.5-6.0)	<0.01*	1.5(0.8-3.2)	0.20
X. Current Alcohol Use						
No		507(75.1)	Reference		Reference	
Yes		168(24.9)	6.6(4.3-8.5)	<0.01*	7.30(3.8-13.8)	< 0.01*
XI. Ever Substance Use						
No		544(80.6)	Reference		Reference	
Yes		131(19.4)	4.8(3.1-7.6)	<0.01*	2.4(1.4-4.3)	< 0.01*

## Discussion

The overall prevalence of non-seat belt use while riding and driving were 19.7% and 19.2%, respectively, in the current study. Among those who rode and drove during the past month, the prevalence of non-seat belt use was 28.03%. The findings in the present study are similar to those reported by Dobhal et al. [8] (Jaipur, Rajasthan; 2019); the prevalence of non-seat belt use was 28% amongst their study participants. In a study conducted by Mukhopadhyay [9] (Panipat, Haryana; 2017), 20.76% of undergraduate college students had not worn a seat belt while driving. Similarly, comparable prevalence rates regarding non-seat belt use have also been reported by Bakar et al. [10] (Turkey, 2020), Kumar et al. [11] (Jammu, 2017), and Ratna et al. [12] (Tumkur, Karnataka; 2017). The current study’s prevalence was lower than the 52% prevalence rate of non-seat belt use reported by Sharma et al. [13] (Delhi, 2008). This difference can be explained by the fact that the mean age of study participants was lower than the mean age in the current study. Furthermore, the study was conducted over 10 years ago. Since then, the relevant authorities have enforced significant policy changes and stringent regulations, such as imposing penalties and fines, to increase compliance with seatbelt use. Massive educational programs and awareness campaigns have also raised public awareness. In the present study, the prevalence of non-seat belt use in riding in a car was higher than the 8.7% reported by Malhotra et al. [14] (Faridabad, Haryana; 2019) amongst young men aged 18-24 in a rural area of North India. This difference can be attributed to the fact that the study populations and settings differed in both studies. The study mentioned earlier was done in a rural area and included participants from only one gender.

In the present study, the prevalence of riding in a vehicle with someone who drank alcohol was similar to the 17% reported by Malhotra et al. [14] (Faridabad, Haryana; 2019) amongst young men in Delhi. However, the prevalence of drinking and driving was reported to be 6% by the authors, which was lower than the 17.2% found in the present study. This difference can be attributed to the differences in the study setting and the socio-economic status of the study participants. Most participants in the current study belonged to urban areas and upper or upper-middle socio-economic class. Peltzer and Pengpid (2015) [15], in their study conducted amongst University students across 26 countries, also reported the prevalence of drinking and driving as 17.3%. However, the prevalence rates in the current study were higher than those observed by Kumar et al. [11] (Jammu, 2017); Nagalingam et al. [16] (Chennai, Tamil Nadu; 2016); Cacodcar and Naik [17] (Goa, 2015); as they reported riding with an intoxicated driver to be 14.7%, 12.5%, and 10.7% respectively. This difference can be due to the different study settings; almost half the participants in these studies belonged to rural areas compared to 25% in the present study. Also, the study population comprised younger participants, with the maximum age reported in all three studies to be only 19 years.

The current study's prevalence rate for using a mobile phone while driving was 37.2%, similar to the findings of the YRBS [7] (CDC, USA, 2019), which reported a prevalence of 39%. It is also comparable to the 40% prevalence reported by Nagalingam et al. [16] (Chennai, Tamil Nadu; 2016) in their study. Further, the current study found that the prevalence of not wearing a helmet amongst those who rode a two-wheeler vehicle in the past 30 days was 42.6%. This finding is similar to the 40% and 46% reported by Kumar et al. [11] (Jammu, 2017) and Abayomi et al. [18] (Nigeria, 2015). The current study also depicts that the odds of indulging in road risk behavior were higher among students who drank alcohol, abused substances, and belonged to a rural area than their counterparts. 

The strengths are the study was conducted across five different administrative zones in Delhi to get a representative sample of the participants. The findings from the present study would contribute considerable input to the policymakers and public health officials for implementing preventive practices. The current study approach can be applied to evaluate and appraise risky road behavioral patterns in various cities across India. This will allow for a more comparative picture and the identification of factors unique to each region.

This article is not without some limitations. First, because the study was cross-sectional, causal inferences cannot be drawn. Second, the survey was conducted among students from a single university in a single state, and including students from other universities in different regions may result in different results. Third, college students are not representative of young adults in general, and road risk behaviors and their risk factors may differ in other population sectors. Fourth, the assessment was based on students' self-reporting, which may have resulted in the under-reporting of risky behaviors due to recall bias. However, a 30-day time frame was implemented to reduce recall bias. Finally, social desirability bias could have occurred because people tend to under-report illegal actions. However, researchers ensured that all identifiers were removed during the survey's design, and participants were informed prior to the survey that their responses could not be traced back to the college or the individual. Nevertheless, the results from the present study are consistent with prior literature published.

## Conclusions

The practice of road risk behaviors was high among college students in Delhi, increasing the likelihood of traffic injuries. The current study also depicts that the odds of indulging in road risk behavior were higher among students who drank alcohol, abused substances, and belonged to a rural area than their counterparts. Based on the findings of this study, we can conclude that comprehensive road safety awareness campaigns, communication strategies, seminars, and workshops aimed specifically at college students must be implemented in all educational institutions. Steps can be taken to educate students about road safety, traffic rules, drunken driving, using mobile phones while driving, and the risks of RTAs. Furthermore, new and innovative strategies must be developed to reduce risky driving behaviors that endanger human life.
